# Do medicine and cell biology talk to each other? A study of vocabulary similarities between fields

**DOI:** 10.1590/1414-431X2021e11728

**Published:** 2021-10-18

**Authors:** S. Azevedo, M.R. Seixas, A.D. Jurberg, C. Mermelstein, M.L. Costa

**Affiliations:** 1 Instituto de Ciências Biomédicas, Universidade Federal do Rio de Janeiro, Rio de Janeiro, RJ, Brasil; 2 Faculdade de Medicina, Universidade Estácio de Sá (Campus Presidente Vargas), Rio de Janeiro, RJ, Brasil

**Keywords:** Cell biology, Medicine, Translational medicine, Text mining, Vocabulary

## Abstract

A close interaction between basic science and applied medicine is to be expected. Therefore, it is important to measure how far apart the field of cell biology and medicine are. Our approach to estimating the distance between these fields was to compare their vocabularies and to quantify the difference in word repertoire. We compared the vocabulary of the title and abstract of articles available in PubMed in two selected high-impact journals in each field: cell biology, medicine, and translational science. Although each journal has its own editorial policy, we showed that within each field there is a small vocabulary difference between the two journals. We developed a word similarity index that can measure how much journals share a common vocabulary. We found a high similarity index between each cell biology (91%), medical (71-74%), and translational journal (65%). In contrast, the comparison between medicine and biology journals produced low correlation values (22-36%), suggesting that their vocabularies are quite dissimilar. Translational medicine journals had medium similarity values when compared to cell biology journals (52-70%) and medicine journals (27-59%). This approach was also performed in 10-year periods to evaluate the evolution of each field. Using the “onomics” strategy presented here, we observed that differences in vocabulary of basic science and medicine have been increasing over time. Since translational medicine has an intermediate vocabulary, we confirmed that translational medicine is an efficient approach to bridge this gap.

## Introduction

Cell biology is one of the major fields of basic science and has been experiencing extremely rapid growth over the past 50 years (1). Medicine uses data originated from cell biology research and other basic science fields to apply the data for the benefit of patients (2-4). It is therefore plausible to expect that the more these two fields exchange information, the greater the benefits for both sides will be. Although this interaction should be intimate, a great divergence has developed between them over the years, and attempts to resolve this have led to the emergence of translational medicine (5-7).

A relevant question, therefore, is how far apart is the vocabulary used in the fields of cell biology and medicine. An easy way to assess the vocabulary of a field is to analyze its article production. Previous attempts have used text mining (8). Such a strategy is now widely used to analyze textual data from scientific literature, especially using article abstracts (9). Some of the approaches involve a large amount of data and computational load (10). One of the main difficulties in this area is the identification of the meaning of a given text and the need to use natural language processing techniques. In search of a simpler alternative that can be applied in several cases without advanced programming, we developed an omics-inspired pipeline that uses freely available tools. Considering that the title and abstract are a condensed representation of concepts and ideas of each article, we hypothesized that the similarity of vocabulary between journals of different fields should be a measure of how related the research fields are. We analyzed the frequency of words in the title and abstract of articles published in leading scientific journals of cell biology and medicine over the past 50 years. While we observed and quantified a small vocabulary difference between articles published in traditional journals in the same field, our findings revealed an ever-widening vocabulary gap between cell biology and medicine. This observation raises important concerns about whether the communication between specialists in different fields hinders the translation of recent advances into clinics, which may be particularly problematic in rapidly evolving situations such as the coronavirus pandemics.

## Material and Methods

### Selection of journals and inclusion criteria

In this study, we compared the vocabulary used in the fields of cell biology, medicine, and translational medicine by analyzing the titles and abstracts of articles published in the Journal of Cell Biology (JCB), Journal of Cell Science (JCS), Lancet (Lan), New England Journal of Medicine (NEJ), Journal of Experimental Medicine (JEM), and Science Translational Medicine (STM). These journals were selected because they represent leading international journals of cell biology (JCB and JCS), medicine (Lan and NEJ), and translational or experimental medicine (JEM and STM) as evidenced by their tradition and high impact factors. It should be pointed out that each has its own editorial policy and that it could be argued that some of them tend to favor particular types of results, such as electron microscopy. We chose to use only the title and abstract because we assumed that they are a concise representation of the full article, because not all full texts were freely available, and because full-text processing can add a lot of extra processing and noise, such as the inclusion of methods and references. Data was retrieved from the PubMed website (https://pubmed.ncbi.nlm.nih.gov/). We excluded Reviews and Editorial Comments from our analysis, so that only regular research articles were included. Our analysis covered approximately the last 50 years (from 1965/75 to 2015), including either all articles or all articles from every two years to fit within the PubMed export size limit of 10,000 articles.

### Data mining and textual analysis

We used the freely available software VOSviewer (https://www.vosviewer.com/) to analyze the frequency of words in titles and abstracts. This information was retrieved from PubMed queries and imported into the VOSviewer, which extracted words from the fields “Title” and “Abstract”. The VOSviewer ignores structured labels and copyright statements. We adjusted the threshold (smallest number of repetitions) to obtain at least 100 words per analysis, since including all words has produced low significance in previous inspections. We chose not to exclude words based on their VOSviewer-computed relevance score (calculated from the assumption that words with high frequency have less “meaning”), since this could lead to the suppression of important words. It is worth noting that VOSviewer removes common, irrelevant English words (that is, “stop words”). We imported all data in Microsoft Excel^TM^ spreadsheets, which we used for further analysis and plotting. To analyze the similarities of word frequencies among journals (“similarity index”), we developed an algorithm that calculated the sum of the frequency of identical words from a given group (a specific journal, for instance) to another group and compared them with the maximum possible identity (100%). To visualize shared words, we used the 40 most frequent words in cell biology and medicine journals to construct a Venn's diagram using a freely available web tool (http://bioinformatics.psb.ugent.be/webtools/Venn/). We created word clouds to represent the proportion of the 100 most frequent words for each journal or period by using the freely available software Wordle (http://www.wordle.net). This graphic representation highlights the most frequent words, which are depicted with a larger font size and a gradient of color. All words were previously lower-cased and stop words were removed.

## Results

Overall, there was a remarkable coincidence in the sequence of the most frequent words between the cell biology journals JCB and JCS (Table 1 and Supplementary Table S1). “Cell” was the most frequent word and “protein” was the second most frequent, probably reflecting the influence of biochemistry in the cell biology field (Table 1). The words “function”, “activity”, “role”, and “mechanism” were also very frequent, suggesting the importance of functional rather than descriptive studies. Next, we analyzed the medical journals NEJ and Lan. “Patient” was the most frequently found word in Lan and NEJ. The higher frequency of “patient” reflects the scope of the medical field, similar to “cell” for cell biology journals. Other very frequent words in Lan and NEJ were “treatment” and “disease”, related to the medical practice (Table 1). Then, we analyzed the frequency of words in the translational medicine journals JEM and STM. Interestingly, in JEM, “cell” was the most frequent word and “mouse” was the second most frequent, while in STM, “patient” was the first and “cell” was the second most frequent word, highlighting the differences of subject in each journal (Table 1). “Disease” and “treatment” were the fourth and fifth most frequent words in STM, which could be expected for a medical journal. These results showed an extremely high coincidence of words within the journals of cell biology and medicine, while the two selected journals of translational medicine displayed less coincidence between them. Overall, we observed a reasonable difference in the vocabulary between journals of cell biology and medicine, whereas journals of translational medicine showed a vocabulary in between that of basic and applied sciences.

**Table 1:** Comparison of the most frequent words in titles and abstracts of articles in cell biology, medicine, and translational research journals.

JCB	JCS	NEJ	Lan	JEM	STM
cell	cell	patient	patient	cell	patient
protein	protein	year	treatment	mouse	cell
function	expression	group	study	t cell	mouse
study	function	percent	group	expression	disease
activity	activity	treatment	year	response	treatment
role	formation	study	trial	antigen	expression
membrane	role	month	disease	study	t cell
formation	effect	rate	month	antibody	study
structure	receptor	number	interpretation	role	model
mechanism	activation	risk	risk	protein	therapy
effect	study	day	data	effect	response
addition	pathway	effect	day	receptor	activity
presence	mechanism	disease	funding	gene	infection
expression	microtubule	age	effect	activity	protein
interaction	interaction	therapy	child	activation	effect
data	structure	use	analysis	mechanism	gene
process	domain	level	use	vitro	development
complex	complex	death	infection	function	tumor
region	process	data	week	data	mutation
site	gene	time	phase	molecule	data

The table only depicts the 20 most frequent words, while the complete data are available in Supplementary Table S1. Color-coded cells represent the same word in different journals. JCB: Journal of Cell Biology; JCS: Journal of Cell Science; Lan: Lancet; NEJ: New England Journal of Medicine; JEM: Journal of Experimental Medicine; STM: Science Translational Medicine.

**Figure 1:**
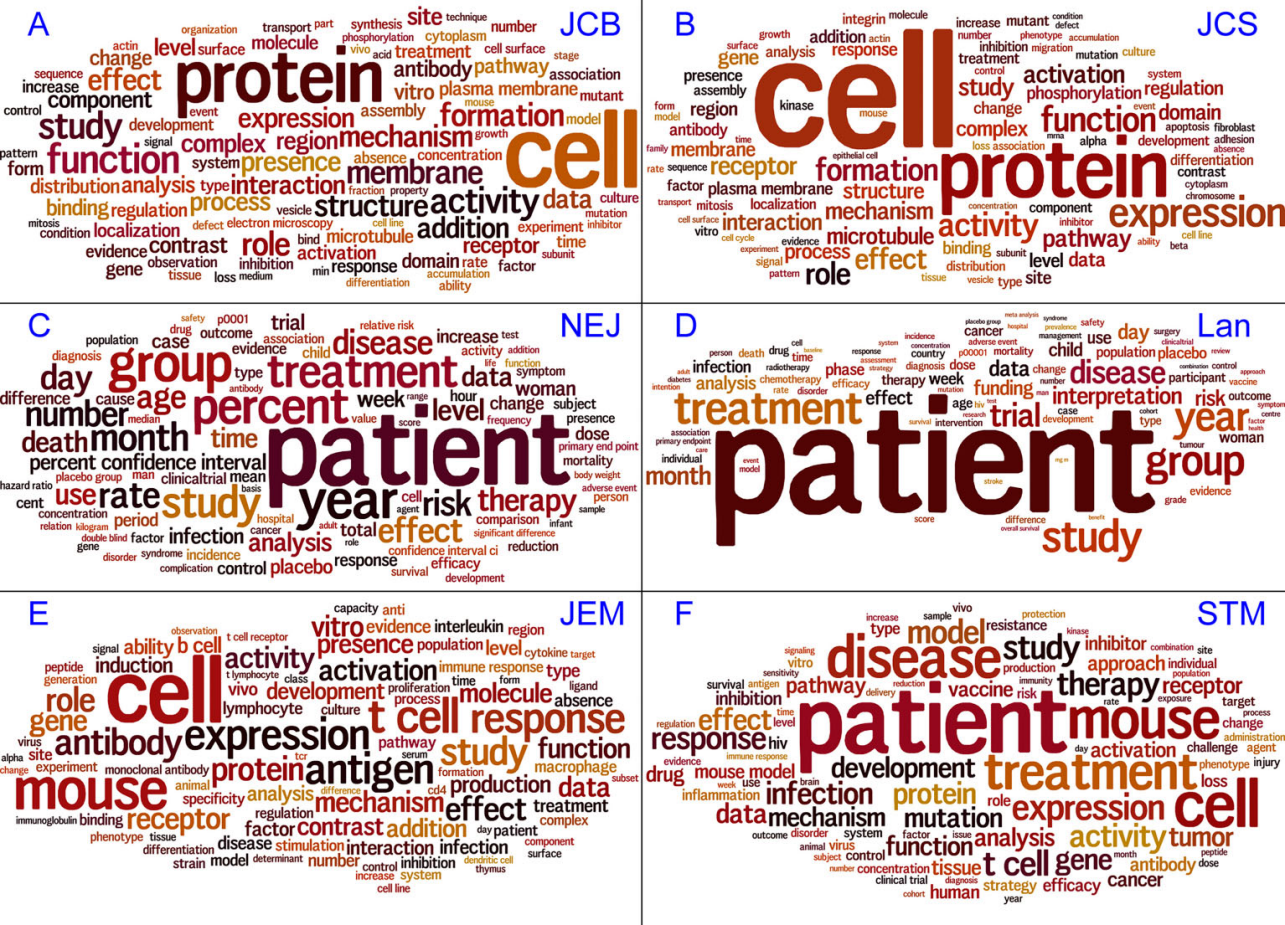
Vocabulary comparison between cell biology, medical, and translational journals using word clouds. There was a remarkable vocabulary similarity between Journal of Cell Biology (JCB) and Journal of Cell Science (JCS), as well as between New England Journal of Medicine (NEJ) and Lancet (Lan). Both Journal of Experimental Medicine (JEM) and Science Translational Medicine (STM) showed a mixture of most frequent words from cell biology and medical journals.

We then used word clouds to better visualize the relative frequency of words in each journal. The images showed that “cell” and “protein” were much more frequent than all other words, both in JCB and JCS, while there was a fairly continuous distribution of word size (indicating the relative frequency) of the rest of the words (Figure 1A and B). In the medical journals NEJ and Lan, the word “patient” stood out (Figure 1C and D), whereas “cell” was the only word in a higher frequency category in JEM and the word “patient” stood out in STM (Figure 1E and F).

**Figure 2:**
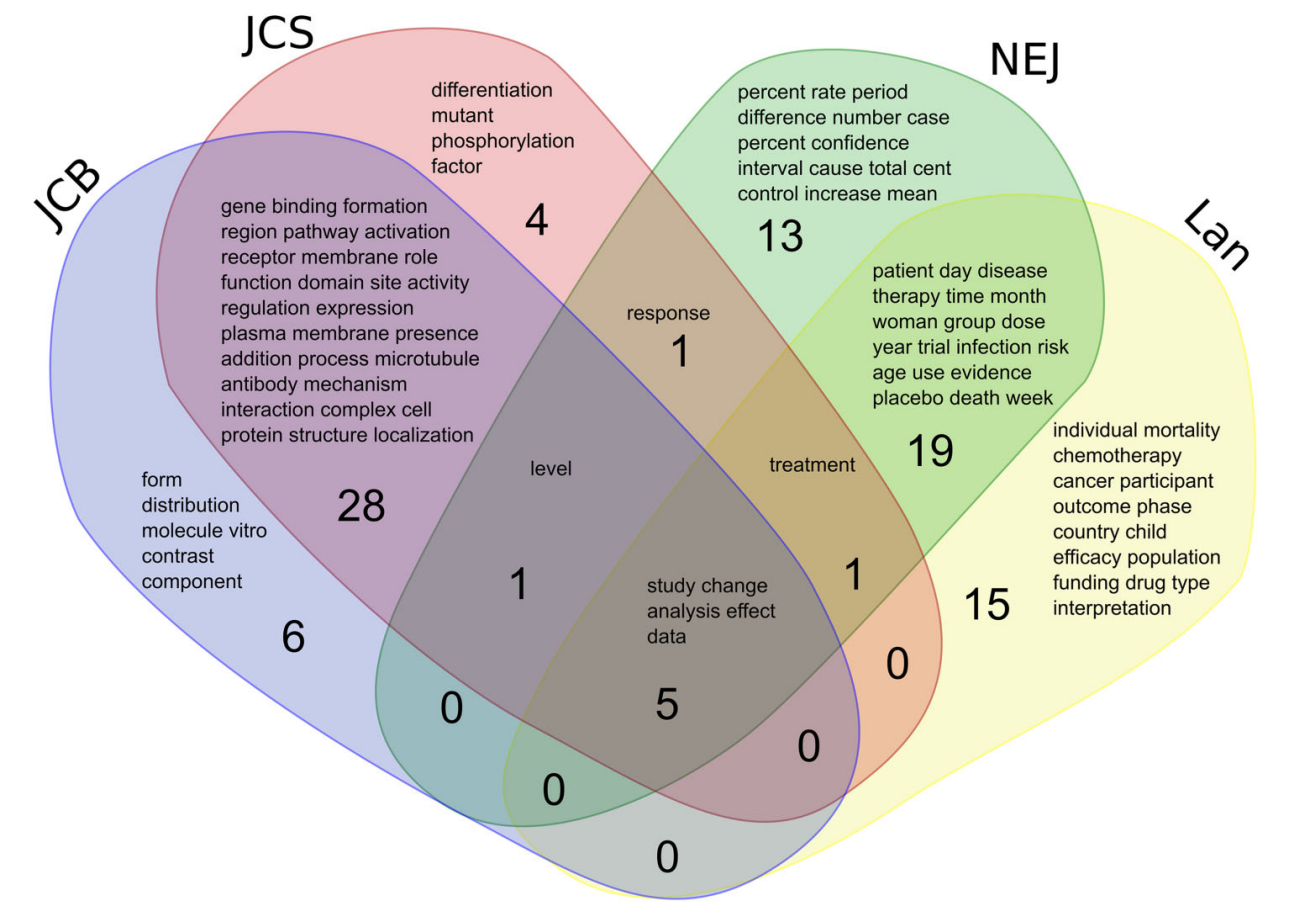
Distribution of highly frequent words in cell biology and medicine journals. We distributed the 40 most frequent words in titles and abstracts of articles from Journal of Cell Biology (JCB), Journal of Cell Science (JCS), New England Journal of Medicine (NEJ), and Lancet (Lan). There was a remarkable vocabulary similarity between JCB and JCS (28 shared words compared to (6+4) unique words), as well as between NEJ and Lan (19 shared words compared to (13+15) unique words). There was a large difference in vocabulary between cell biology and medicine journals, with only 5 words in common.

To analyze the shared use of these high frequency words in cell biology and medical journals, we constructed a Venn diagram with the 40 most frequent words. Only five words were found in common between the four journals (Figure 2), as opposed to a large number of words that were used simultaneously in the two cell biology journals (28 same words in JCB and JCS) and in the two medical journals (19 same words in Lan and NEJ). Interestingly, no words were found in common between JCB and Lan, JCB and NEJ, JCS and NEJ, or JCS and Lan (Figure 2).

While the Venn diagram identified shared words, it did not take into consideration the relative frequency of each word. For this reason, we developed in a spreadsheet an algorithm to compare the relative frequency of words (“similarity index”) from journal pairs (Table 2 and Supplementary Table S1). In the spreadsheet, we listed the 100 most common words for each journal with their frequencies. We then analyzed for each spreadsheet cell if its content was found in the whole list of words of a journal: if the word was repeated, its frequency was retained, otherwise the frequency was taken as zero. Afterwards, the algorithm calculated the sum of the retained frequencies and compared this sum with the maximum identity value (100%). Since the total number of words varied from journal to journal, the similarity index differed between two journals depending on which journal was used as the source for the original list of words to be compared (for instance: the similarity index of JCB compared to NEJ was 35, while the index of NEJ compared to JCB was 29). We found extremely high similarity indices between JCB and JCS, as well as between Lan and NEJ (Table 2, in bold type). JEM and STM showed slightly smaller similarities. Conversely, we observed lower values of similarity between cell biology and medicine journals (Table 2, in italic type). In turn, JEM exhibited a vocabulary more similar to cell biology journals (similarity indices between 67-70) than to medical journals (values between 27-38), while STM vocabulary was equally shared with cell biology (values between 52-57) and medicine (values between 53-59). These observations indicated that one of the selected journals of translational medicine showed more similarities with cell biology journals than with medical journals. Overall, our findings revealed striking differences in the vocabulary used in the cell biology and medicine research fields, thus pointing out the gap between these two major fields.

**Table 2:** Similarity indexes between vocabulary of title and abstracts of articles from cell biology, medicine, and translational journals.

	Cell biology		Medicine		Translational medicine	
	JCB	JCS	NEJ	Lan	JEM	STM
JCB	100	**91**	*35*	*25*	69	52
JCS	**91**	100	*36*	*26*	70	57
NEJ	*29*	*29*	100	**71**	38	57
Lan	*22*	*22*	**74**	100	37	59
JEM	67	67	38	27	100	65
STM	52	52	53	53	65	100

Numbers in bold correspond to the similarity between the two cell biology journals and between the two medicine journals, while numbers in italics correspond to the similarity between cell biology and medicine journals. The complete table with the calculations is available in Supplementary Table S1. JCB: Journal of Cell Biology; JCS: Journal of Cell Science; Lan: Lancet; NEJ: New England Journal of Medicine; JEM: Journal of Experimental Medicine; STM: Science Translational Medicine.

Next, we evaluated the vocabulary changes every 10 years over a 50-year period to address the evolution of each field. In all the periods studied of both cell biology journals, “cell” was the most frequent word (Supplementary Figure S1). The word “protein” was in second position since 1985 in JCB and since 1995 in JCS. “Membrane” changed from a highly frequent word in the years 1965-1985 to less frequent from 1995-2015 in JCB and JCS. Another important change was the replacement of words related to the structural description of organelles (such as “structure”, “region”, “cytoplasm”, and “electron microscopy”) in the earlier years of JCB and JCS to dynamic cellular processes (such as “regulation”, “activation”, “mechanism”, “expression”, “role”, “pathway”, and “function”) in more recent years (process-related words are shown in bold). In both medical journals, “patient” was among the most frequent words in all years. The words “treatment”, “disease”, and therapy” were very frequent in Lan and were still very frequent in NEJ, though less so than in Lan. In the medical journals, we observed a high frequency of method-related words, such as “age”, “year”, and “percent”. We did not observe a clear tendency of word substitutions over time as observed in cell biology journals. Since STM started to be published in 2009, our analysis of the translational medicine journals was performed only with JEM. “Cell” was the most frequent word in JEM in all periods studied, similar to the pattern observed in the selected cell biology journals. “Mouse”, “antibody”, and “antigen” were also highly frequent in JEM. Similar to cell biology journals, the word “mechanism” has recently increased in frequency.

**Figure 3:**
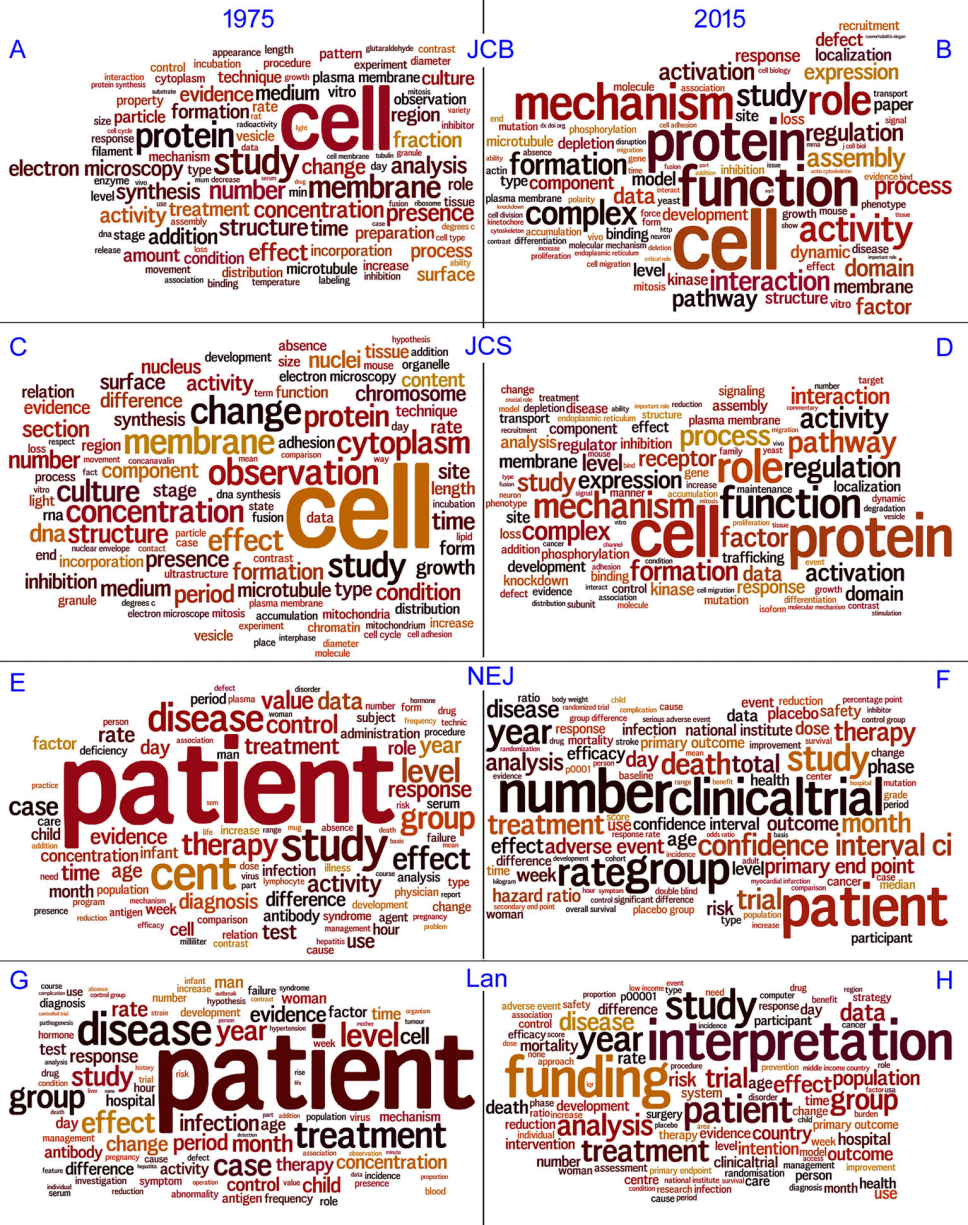
Vocabulary comparison over time in cell biology and medical journals using word clouds. The vocabulary of cell biology journals changed more over time than of the medical journals. JCB: Journal of Cell Biology; JCS: Journal of Cell Science; Lan: Lancet; NEJ: New England Journal of Medicine.

To better visualize the relative frequency of words in each journal over time, we again used word clouds. “Cell” was by far the most frequent word in 1975 and in 2015 in both JCB and JCS (Figure 3A-D). The word “protein” increased in frequency over time until it became highly frequent. The words “function” and “mechanism” were very frequent in 2015, while they were absent from the top 20 words in 1975. In the medical journals Lan and NEJ, the word “patient” was by far the most frequent word in 1975, but its frequency decreased in 2015, more evidently in Lan than in NEJ (Figure 3E-H). Other words, including the method-related words “number”, “clinical trial”, “interpretation”, and “funding” increased in frequency over time.

To quantify the changes in vocabulary similarities over time, we generated a similarity index comparing the early and late years of cell biology and medical journals (Table 3 and Supplementary Table S2). The vocabulary of cell biology journals was already similar in early years and similarity increased even more over time, while the vocabulary of the medical journals was similar in early years and similarity slightly decreased over time. In turn, similarity between cell biology and medical journals showed a great reduction over time (Table 3). Importantly, the low similarity between fields in the early years became even lower in recent years, suggesting that the vocabulary gap between the basic cell biology and applied biomedical fields has widened over time.

**Table 3:** Similarity indices between vocabulary in titles and abstracts of articles from cell biology and medicine journals between the years 1975 and 2015.

	Cell biology				Medicine			
	JCB		JCS		NEJ		Lan	
	1975	2015	1975	2015	1975	2015	1975	2015
JCB 1975/2015	100	100	69	86	38	12	39	18
JCS 1975/2015	67	81	100	100	34	18	34	23
NEJ 1975/2015	35	12	28	22	100	100	79	74
Lan 1975/2015	36	17	27	25	84	66	100	100

The complete table with the calculations is available in Supplementary Table S2. All word lists were compared to each other, but the table depicts only the comparisons between the same years for each journal (i.e., JCB 1975 × JCS 1975, JCB 2015 × JCS 2015, etc). JCB: Journal of Cell Biology; JCS: Journal of Cell Science; Lan: Lancet; NEJ: New England Journal of Medicine.

## Discussion

We identified a striking similarity between the vocabulary in titles and abstracts of articles between two leading cell biology journals and between articles from two different leading medical journals. In contrast, the vocabulary of cell biology articles was quite different from that of medical articles. More importantly, we were able to measure these similarities and differences. The gap between basic science and medicine vocabulary has already been identified by several authors (5-7). We quantified this gap and showed that not only was it huge by 1975, but the gap had widened over the last 50 years. At the same time, we analyzed two translational medicine journals (a traditional one and a newer one) whose vocabulary fitted reasonably in between both fields. It is therefore reasonable to assume that they represent the growth in translational biomedical science to bridge this gap.

We decided to use only the title and abstract of the articles because of their free availability and simplicity. Abstracts should reflect a selected and meaningful repertoire of words used in the full text. The correspondence of vocabulary between abstract and text has been demonstrated previously (10). However, some authors have argued that parsing full texts would be more informative (11). Although we concur that mining full texts could provide valuable additional information, as in the case of the identification of relationships between proteins and genes, there are many situations where parsing full texts could prove troublesome or misleading (11), not to mention more expensive (if a commercial software is used). More specifically, it is not clear what parts of the full texts should be used: it may be reasonable to exclude references and even Materials and Methods. Indeed, our preliminary results suggested that the analysis of abstracts is, in some situations, more meaningful than using the full text (data not shown). Such hindrances could be bypassed by text cleaning techniques and advanced lexicometric analyses using natural language processing and special techniques, such as stemming and lemmatization, but this may not be accessible to health researchers or may imply the involvement of computer science experts. Our proposed pipeline takes advantage of freely available, easy-to-use tools and involves no prior knowledge of programming. Importantly, by using comparative journals as controls, we showed that the analysis of word frequencies in titles and abstracts provided a simple but powerful and quantitative approach to evaluate the vocabulary proximity and evolution between fields such as cell biology and medicine.

In the present study, we used two leading journals of each field (cell biology, medicine, and translational studies) to comparatively analyze vocabulary frequencies. We obtained robust results, i.e., large similarity indices within each field, small similarity indices between the basic and applied sciences, and intermediate values between translational sciences and both basic and medical fields. In future studies, we intend to expand our database using a larger number of journals to further characterize each field and to compare them with other fields, such as biochemistry.

Not only is it reasonable to assume that the vocabulary of major journals reflects the general interests of respective fields, but our observation that two journals in each field also behave in a similar way suggests that our results are not limited to a particular editorial policy. One result that called our attention was the large change over time in the vocabulary of cell biology articles compared to a small change in the medical articles. Cell biology seems to have changed progressively from a descriptive vocabulary in 1965 into a mechanistic one in 2015. Interestingly, Sato and Sato (12) studied the history of biology from PubMed articles of 32 selected journals from 1965 to 2014 and they found similar results. They suggest that function-oriented studies are a new trend of biologists in the genomics era after 1997, in which biological research focused on identifying a link between a molecule or a structure with its function. In agreement with our results, Mayor (13) observed a dramatic change in the language used in cell biology today compared to the 1950s and 1960s. He attributed the change to the fact that cell biology has moved into a truly interdisciplinary outlook at the interface of biophysics, biochemistry, mathematics, and genetics.

It is interesting to compare our vocabulary analysis to the study of plagiarism, which has become a major publishing concern (14). Plagiarism can range from the more easily detectable literal copy of sentences to the more difficult to identify copy of concepts or ideas. Plagiarism detection algorithms also measure the similarity between texts, as we did, but they focus on specific word sequences in documents (15), while in our vocabulary analysis, the word sequence was irrelevant.

The way we analyzed words could be compared to two major fields of study: linguistics, including language evolution, and genomics, particularly population analysis. Since our background is in biological sciences, we will not discuss our results in the context of linguistics. Our approach is quite similar to the omics studies, where the frequencies of genes, transcripts, or proteins of different groups (control × treated, population A × population B) are compared. The assumption in omics studies is that there are either evolutionary or adaptive/functional reasons for the observed differences, but the first step in identifying the differences in group frequencies does not take into account the need to understand such underlying reasons. For instance, the analysis of single nucleotide polymorphisms (SNPs) in genomes of separated groups has demonstrated that they are useful tools to quantify differences among populations and predict disease outcome: SNPs are used because they are easy to identify and they do not have any meaning per se (16). In this respect, it is expected (but not necessarily true) that most, if not all, high frequency words actually reflect the relative importance of concepts at a given time. This is actually a testable hypothesis, since we can assign an adaptive value to each journal, based on its impact factor, and compare its vocabulary to that of other journals. We should even be able to test if there is a relationship between the number of citations of a given article and its similarity to the area archetype.

Since our approach uses words in a similar way to genomics, we propose the new term “onomics” (from the Greek “onome” = word) to describe the way we analyze vocabulary. Our study could be considered part of memetics, the analogy between genes and concepts created by Dawkins, when we assumed that every word is a meme: “Everything that is passed from person to person in this way is a meme. This includes all the words in your vocabulary, the stories you know,…” (17). Accordingly, we might call our approach “memomics”. In this analogy to memes, the “replicators” are the articles and journals. While Dawkins assumed that each gene has its own (“selfish”) adaptive advantage, and that the whole genome (or a cell or an individual) is not the main level of selection, he also acknowledged the existence of groups of genes that are selected together (memeplexes) (18).

## Supplementary Material

Click here to view [zip].
